# The Surgery of Western Nations

**Published:** 1860-01

**Authors:** 


ART. X.—*The Surgery of Western Nations.* By H. HOBSON, M.D., London Mission Society. Shanghae, 1857.

A WORK on surgery, published in the Chinese language, is a curiosity. That a Christian dog should have ventured upon such an enterprise must have greatly surprised the Celestials, for whose special benefit the book has been prepared. Dr. Hobson deserves well of these people for his attempts to enlighten them upon professional matters. The "Surgery of Western Nations" is intended as a companion to his works on anatomy, physiology, chemistry, midwifery, and practice of medicine, which are all issued in the same uniform style. Our Chinese scholarship is not very profound, but we *guess* that the letter-press is well executed, while, if we are any judge of the picturesque, we have no hesitation in saying that the wood-cuts which accompany these productions are utterly destitute of artistic finish. Indeed, nothing could be more crude and imperfect. Those in the treatise on surgery, upwards of 400 in number, are copied from Liston, Ferguson, Erichsen, Miller, Druitt, and Wharton Jones, and exhibit a very odd, dislocated appearance among the Chinese hieroglyphics, whose meaning they are intended to elucidate.

We are indebted for copies of these works to our collaborator, Dr. John G. Kerr, of Canton, whose article on "Medicine in China," in a former number of the Review, attracted so much attention. Our friend is doing good service at the Missionary Hospital of that city, and we trust that his Celestial Majesty may be induced, one of these days, to make him his privy counselor and surgeon in ordinary.

